# Permissible Home Range Estimation (PHRE) in Restricted Habitats: A New Algorithm and an Evaluation for Sea Otters

**DOI:** 10.1371/journal.pone.0150547

**Published:** 2016-03-22

**Authors:** L. Max Tarjan, M. Tim Tinker

**Affiliations:** 1 Department of Ecology and Evolutionary Biology, University of California Santa Cruz, Santa Cruz, California, United States of America; 2 Western Ecological Research Center, U.S. Geological Survey, Santa Cruz, California, United States of America; Hokkaido University, JAPAN

## Abstract

Parametric and nonparametric kernel methods dominate studies of animal home ranges and space use. Most existing methods are unable to incorporate information about the underlying physical environment, leading to poor performance in excluding areas that are not used. Using radio-telemetry data from sea otters, we developed and evaluated a new algorithm for estimating home ranges (hereafter Permissible Home Range Estimation, or “PHRE”) that reflects habitat suitability. We began by transforming sighting locations into relevant landscape features (for sea otters, coastal position and distance from shore). Then, we generated a bivariate kernel probability density function in landscape space and back-transformed this to geographic space in order to define a permissible home range. Compared to two commonly used home range estimation methods, kernel densities and local convex hulls, PHRE better excluded unused areas and required a smaller sample size. Our PHRE method is applicable to species whose ranges are restricted by complex physical boundaries or environmental gradients and will improve understanding of habitat-use requirements and, ultimately, aid in conservation efforts.

## Introduction

Home range estimates are useful tools for answering critical questions in studies of habitat selection [1–3], mating systems [4,5], and carrying capacity [6], and in identifying the biotic and abiotic features with which individuals interact. Statistical methods for estimating home ranges, defined as the particular area to which an animal restricts its movements over time [7,8], use sighting locations to estimate the probability of an individual occurring at any point in space, and to delineate a boundary encompassing some cumulative probability of occurrence. These boundaries denote the location, shape, and size of the home range [9].

A notable limitation of existing methods is their inconsistent performance across species and habitats [10,11]. Current methods typically perform well for animals that make indiscriminant use of open, uniform habitats, but poorly for animals that concentrate space use around patchy resources (e.g., tawny owls [*Strix aluco*] in woodland patches [12] and red-capped robins [*Petroica goodenovii*] in woodland remnants [13]) or are restricted by complex boundaries [6] (e.g., river otters [*Lontra candensis*] at the aquatic-terrestrial interface [14], flathead catfish [*Pylodictis olivaris*] in streams [15], weasels [*Mustela nivalis*] inhabiting field edges [16], and raccoons [*Procyon lotor*] along habitat edges [17]). In the latter case, existing methods generally fail to exclude unusable habitat [18–21]. This positive bias (type II error) causes an overestimate of home range area and affects our ability to understand patterns of resource use and exposure to sources of mortality.

Current methods derive home range estimates from sighting locations anchored in “geographic space,” generally defined using a two-dimensional Cartesian coordinate system such as latitude and longitude. However, animal space use is often determined by ecological characteristics, which are defined by environmental features and/or the space use of conspecifics and other species. Hence, there is often a mismatch between how we estimate home ranges and how animals actually choose their home ranges, so we should not be surprised that our methods sometimes perform poorly. A number of modifications to traditional home range analyses have been proposed to address this issue; for example, Horne et al. [22] created a synoptic model of animal space use that produces home ranges informed by habitat covariates. Although this method can be applied to animals whose home ranges track a linear habitat feature [23], the accuracy of the so-called “null distribution” of the synoptic model decreases as the linear feature becomes more tortuous. Take, for example, the case of a strictly marine species that uses one side of a peninsula. Although the presence of water (1) or land (0) can be added as a binomial habitat covariate, the null distribution will cause points on one side of the peninsula to influence probability values on the other side of the peninsula, even if the animal is only found on a single side. This is because the two sides of the peninsula are close to each other in geographic coordinate space, but are far apart from the perspective of an animal traveling through the water. Home range analysis in such cases remains problematic; in particular, it remains difficult to derive home range estimates that do not violate known habitat restrictions for species with tortuous linear boundaries in their habitat.

Mechanistic movement models offer a sophisticated method for incorporating environmental covariates (e.g. coyote prey and conspecific scent marks [24]) into animal movement decisions. Home range estimates can be derived from such movement models, as employed in Mechanistic Home-Range Analysis (MHRA) [24]. This powerful approach confers the ability to predict future movements, but may be unnecessarily complex for obtaining descriptions of past home ranges. As pointed out in Moorcroft’s review [9], MHRA is challenging to implement as it requires programming expertise, is computationally expensive, and/or requires familiarity with formulating and solving systems of differential equations. These challenges may explain the persistent and widespread use of simpler descriptive methods of home range estimation. We aimed to provide an accessible method with the explicit purpose of describing the observed space use of an animal.

As with many coastal species, sea otter (*Enhydra lutris*, Linnaeus 1758) space use is restricted by the complex coastal boundary and the heterogeneous habitats that characterize nearshore environments. Sea otters live predominantly in aquatic areas, and avoid hauling out more than a few meters inland due to poor motility on land, vulnerability to terrestrial predators, and their requirement for frequent feeding bouts in the ocean. In addition, physiological limits in diving capabilities restrict sea otters to areas where benthic invertebrate prey are accessible (generally <40 meters depth) [25,26]. Despite the >76,000 radio-telemetry sighting locations of individual sea otters collected to date from studies in central California [27] and the wealth of knowledge about sea otter habitat requirements, a suitable home range estimator is lacking. Home ranges created using kernel density estimation (KDE) [28,29] and adaptive Local Convex Hull analysis (a-LoCoH) [19] typically overlap with terrestrial areas that are too far inland to be accessible to sea otters and/or include aquatic areas that are too far offshore or too deep, leading to incorrect expectations about access to resources and home range size and shape. To address this problem in sea otters and other species with similar restrictions to movement, we present a novel analysis that incorporates features of habitat suitability (boundaries and environmental gradients) into home range estimates (i.e. geographic ranges of animals in space).

Using radio-telemetry data on sea otters, we develop and evaluate a method for estimating permissible home ranges (hereafter “Permissible Home Range Estimation,” or PHRE) that: (1) reflects ecological and physiological constraints on animal movements, (2) generates probability estimates based on habitat suitability, and (3) produces robust, unbiased estimates of the areas actually used by individual sea otters. We describe a generalized function for PHRE coded in the open source statistical program R [30]. In addition, we evaluate and compare sample size requirements and the predictive accuracy of probability estimates of PHRE and two commonly used methods: kernel density estimation and Local Convex Hull Analysis. We then use this new method to test the prediction that resource distribution across sites in central California affects the shape of sea otter home ranges.

## Methods

### Ethics statement

This research adhered strictly to established capture, tagging, and tracking protocols, which were reviewed and approved by the University of California Santa Cruz Institutional Animal Care and Use Committee and the U.S. Fish and Wildlife Service. Protocols were conducted under the following research permits: University of California Santa Cruz IACUC permit Tinkt1007 (8/05/2010) and U.S. Fish and Wildlife Service permit MA672624-16 (10/31/2008). As outlined and approved in the established protocols, animals were sedated for surgery with an intramuscular injection of fentanyl (Elkins-Sinn, Cherry Hill, NJ, USA; 0.5–0.11 mg kg^−1^ body mass) and diazepam (Abbot Laboratories, North Chicago, USA; 0.010–0.053 mg kg^−1^) and maintained under an isoflourane gas and oxygen mixture [31].

### Data collection

From 1999 to the present, U.S. Geological Survey and Monterey Bay Aquarium scientists and volunteers collected spatially explicit sighting data from radio-tagged sea otters near Monterey Bay (36.6183° N, 121.9015° W) and Big Sur, California (36.1075° N, 121.6258° W). We captured sea otters using rebreather SCUBA and Wilson traps [32], surgically implanted them with VHF radio transmitters (Advanced Telemetry Systems Inc., Isanti, MN, USA), and applied color-coded plastic flipper tags in the webbing of the hind flippers (Temple Tags, Temple, TX, USA) to aid in visual identification [31,33].

We visually located tagged individuals during regular field surveys (usually 3–5 times per week, but less often for some wide-ranging individuals) using standard VHF radio telemetric techniques [34,35] for multiple years. This resulted in 38,941 sighting locations for 193 individuals. While autocorrelation is of concern in home range estimation, sea otters routinely travel the full length of their home range in a single day, so we treated the sighting locations (which are only collected every few days) as independent. Associated observational data collected at the time of each sighting confirmed that all sighting locations were in water, and that terrestrial areas represented unused habitat. Sighting locations from 126 sea otters with >20 sighting locations per individual over a two-year period were used to compare the performance of three home range estimation methods. Because home range boundaries often change over an animal’s lifetime (e.g. male sea otters disperse as juveniles and may settle into small reproductive territories as adults [34]) and comparisons are meaningful only if home ranges are calculated over the same time period [10], we used two years of data for each home range estimate.

### Model Description

PHRE consists of four steps: (1) identify habitat elements that influence animal space use a priori, (2) transform sighting locations to a new coordinate system reflecting key habitat variables, (3) produce a kernel density estimate in landscape space, and (4) back-transform the KD probability values to geographic coordinate space. For sea otters that move primarily up and down the coast within the nearshore environment, the key habitat elements defining space use are position along the California coastline and distance from shore (S1 Fig). Coastal position in California is easily described by a previously-defined one-dimensional axis termed the “As The Otter Swims” (ATOS) line, representing a sequentially numbered set of points at 500-m intervals along the 10-m isobath [36] (S2 Fig). Each sighting location was transformed to decimal ATOS units by linear interpolation (e.g. a sighting location that was 1/3 of the way between ATOS point 367 and 368 was assigned a value of 367.33). The perpendicular distance to the closest shoreline feature was also calculated for each sighting location using the 1:24,000 coastline vector [37]. The resulting transformed coordinate system better reflected movement decisions by the animal (i.e. animals decide to move up or down the coast, and on or off shore), and also flattened out the tortuous linear boundary.

We further transformed the distance-from-shore values to ensure complete exclusion of terrestrial areas from home range estimates and to normalize the right-skewed distribution. Specifically, we log-transformed the raw distance-from-shore values (S3 Fig), which resulted in a distribution that was approximately normal, varied in log-space from –∞ to ∞ and, importantly, prohibited assignment of probability values >0 on land because log(0) is undefined. We note that for boundaries having an environmental value other than 0, or for environmental variables where log-transformation is not appropriate, an alternative approach is to use a truncated normal distribution for the kernel along the target axis. These practices are key to complete exclusion of unused areas when a distinct boundary exists, especially if the animal heavily uses areas immediately adjacent to the boundary.

We next fit a bivariate kernel density function (ks package [38] in R version 3.0.2 [30]) using the decimal ATOS and *log*(distance) variables for each individual (S4 Fig). Otters are known to differ in the nature of their coastal movements, with some individuals (e.g. adult females) making small movements and using a highly concentrated area of coast, and other individuals (e.g. juvenile males) making longer movements and utilizing large areas of the coast [39]. To account for these different space-use patterns we used an adaptive smoothing parameter (*h*) for the decimal ATOS axis. We allowed the value of *h* to vary as a function of the mean nearest neighbor distance (*d*) between sighting locations, according to the equation *h* = *h*_*b*_·(*d*/4)^2.5^, where *h*_*b*_ represents the baseline smoothing parameter of 2 ATOS units, or 1 km of coastline. This equation was selected prior to home range analyses using a subset of animal location data and based on subjective visual choice [28,40,41] of a parameter that consistently avoided under- and over-smoothing for a variety of different movement types (i.e. those represented by adult females, juvenile males, and adult males). The smoothing parameter for the *log*(distance) axis was held fixed at 0.05.

**Fig 1:**
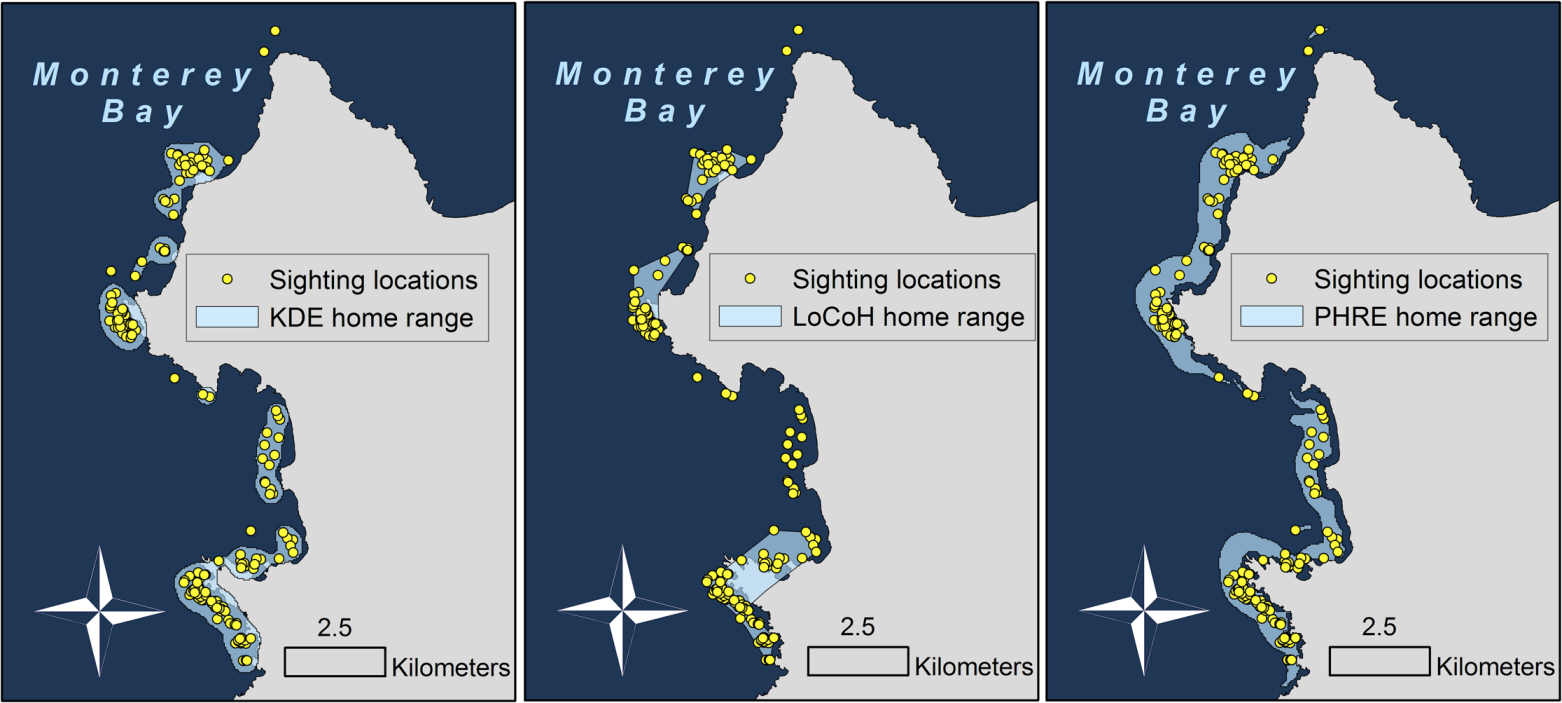
Home range polygons estimated using three different methods. The polygons represent the 90% probability isopleth of sea otter number 1392, a female in Monterey Bay, CA. Note the overlap between home range polygons and land for LoCoH (middle; 17% of the home range overlapped with land) and KDE (left; 10%), but not for PHRE (right; 0%).

The kernel density function was then back-transformed to geographic space by evaluating the probability density values across a grid of points with local coverage (S5 Fig). All density values in the grid were then transformed to sum to one and reflect probability values. A polygon was created to encompass grid points within the 90% kernel home range boundaries (Fig 1 & S6 Fig). We created a function that applies the above-described algorithm to any dataset, using the open source statistical programming language R (version 3.0.2) [30] and incorporating the ks [38], raster [42], and amap [43] packages (S1 File). This generalized function requires sighting locations and a list of raster datasets with habitat elements of interest (the analysis handles one to six dimensions in landscape space). Optional specifications include the percent kernel and the smoothing parameter.

### Method Comparison

We evaluated the method’s utility by comparing its general performance with two other commonly used methods of estimating home ranges in geographic space: (1) kernel density estimation (KDE) [28,29] and (2) adaptive Local Convex Hull analysis (a-LoCoH) [19], a nonparametric method designed to delineate habitat boundaries. We used the ks package [38] for KDE and the adehabitatHR package [44] for LoCoH. The baseline smoothing parameter was 30,000 for KDE, which we then adapted for each animal using the same method as applied in PHRE. As suggested by Getz et al. [20], we used the maximum distance between sighting locations of the animal being evaluated for the LoCoH smoothing parameter. All three methods therefore adapted the smoothing parameter according to the distribution of sighting locations for each animal. We selected 90% isopleths as home range boundaries (Fig 1). The performance of each method was then evaluated based on the following metrics: (a) the ability to exclude unused (terrestrial) areas from home range estimates, (b) the minimum sample size of sighting locations required, and (c) the predictive accuracy of probability estimates.

#### Exclusion of unused areas

To test the ability of each method to exclude terrestrial areas, we calculated the percent of home range area that overlapped with terrestrial habitat (1:24,000 shoreline feature) using the rgeos package [45] in R version 3.0.2 [30].

#### Sample size requirement

We iterated home range estimates across different sample sizes of sighting locations (*N* = 10 to 300 in increments of 10) using data for 26 animals with ≥300 sighting locations within a two-year period. For each sample size, we subsampled data without replacement ten times. We identified the minimum sample size requirement (defined as the minimum *N* that produced mean home range areas statistically similar to the estimated area at *N* = 300) [46] for each animal using a Kruskal-Wallis test and Wilcoxon rank sum tests with a Bonferroni correction (adjusted cutoff value at *p* = 0.0167) across sample sizes (data were non-normally distributed, Kolmogorov-Smirnov test, *p* << 0.05) and compared requirements across methods. To address variation around mean area, we fit an asymptotic curve to the coefficient of variation (CV) and determined at what *N* the CV reached an asymptote. We compared CV sample size requirements across methods using a one-way analysis of variance (ANOVA) executed with the aov function (data were normally distributed within methods, Kolmogorov-Smirnov test, *p* >> 0.05).

#### Predictive accuracy of probability estimates

For 26 animals with ≥300 sighting locations, we generated home range estimates for each method using 200 sighting locations (greater than the maximum sample size requirement for all methods; see results below). Rather than restrict estimates to simplified 90% isopleth boundaries, we tested the predictive accuracy of the probability grids from PHRE and KDE and created probability grids for LoCoH using the 10–100% isopleths (at 10% intervals). We evaluated all methods on their ability to predict the location of 100 randomly selected sighting locations (presence points)—independent from those used for the home range estimate—against a background sample of 1,000 pseudo-absence points, which were randomly drawn from within 1 km of the sighting locations. We iterated this process ten times and calculated the average area under the receiver operating characteristic curve (AUC) [47–49] for each method using the dismo package [50] in R. AUC is a threshold-independent measure of model performance that calculates the proportion of pixels correctly or incorrectly classified. The receiver operating characteristic curve depicts the relationship between the proportion of correctly predicted presences (i.e. the true positive rate) against the proportion of incorrectly predicted absences (i.e. the false positive rate). AUC ranges from 0.5 for a model that is no better than chance to 1.0 for a model that perfectly predicts presences and absences. AUC values did not meet assumptions of a normal distribution (Kolmogorov-Smirnov test, *p* < 0.05), so we compared across methods using a Kruskal-Wallis test and Wilcoxon rank sum tests with a Bonferroni correction (adjusted cutoff value at *p* = 0.0167).

### An Ecological Application

In addition to comparing the methods, we used all three home range estimators to test predictions about the effects of habitat structure on sea otter space use. Home ranges should result from animals maximizing benefits—resources contained within an area—while minimizing costs of travel and resource extraction [6], so size and shape should be influenced by resource availability and distribution. Due to physiological limits in sea otter diving capabilities [25,26], the continental-shelf extent has a large impact on offshore availability of benthic prey. Among study sites for this project, Monterey Bay has a more extensive continental shelf than Big Sur, so sea otters are capable of accessing prey resources farther offshore in Monterey Bay. We hypothesized that size and shape of home ranges are influenced by these differences in resource distribution between sites.

We quantified the amount of available habitat in Monterey Bay and Big Sur, CA based on coastal bathymetry. We used 200-m resolution bathymetry data [51] to select areas that are accessible to diving sea otters (0 to −39 m depth, which encompasses the 99^th^ percentile of diving depths for sea otters in Monterey Bay and Big Sur [27]). We tested our hypothesis that habitat bathymetry affects home range shape by plotting home range length (the distance along the “as the otter swims” line [10-m isobath] encompassed within the home range polygon) vs. home range area—where slope represented the length-area relationship (i.e. home range shape)—and comparing the slopes between sites. Higher slopes in this case indicate more elongated home range polygons. We compared the ability of each method to detect differences in home range shape between sites by evaluating the assumption of homogeneous slopes of fitted, log-linearized functions using analysis of covariance (ANCOVA; data were normally distributed; Kolmogorov-Smirnov test, *p* > 0.1) [30].

## Results

### Method Comparison

#### Exclusion of unused areas

**Fig 2:**
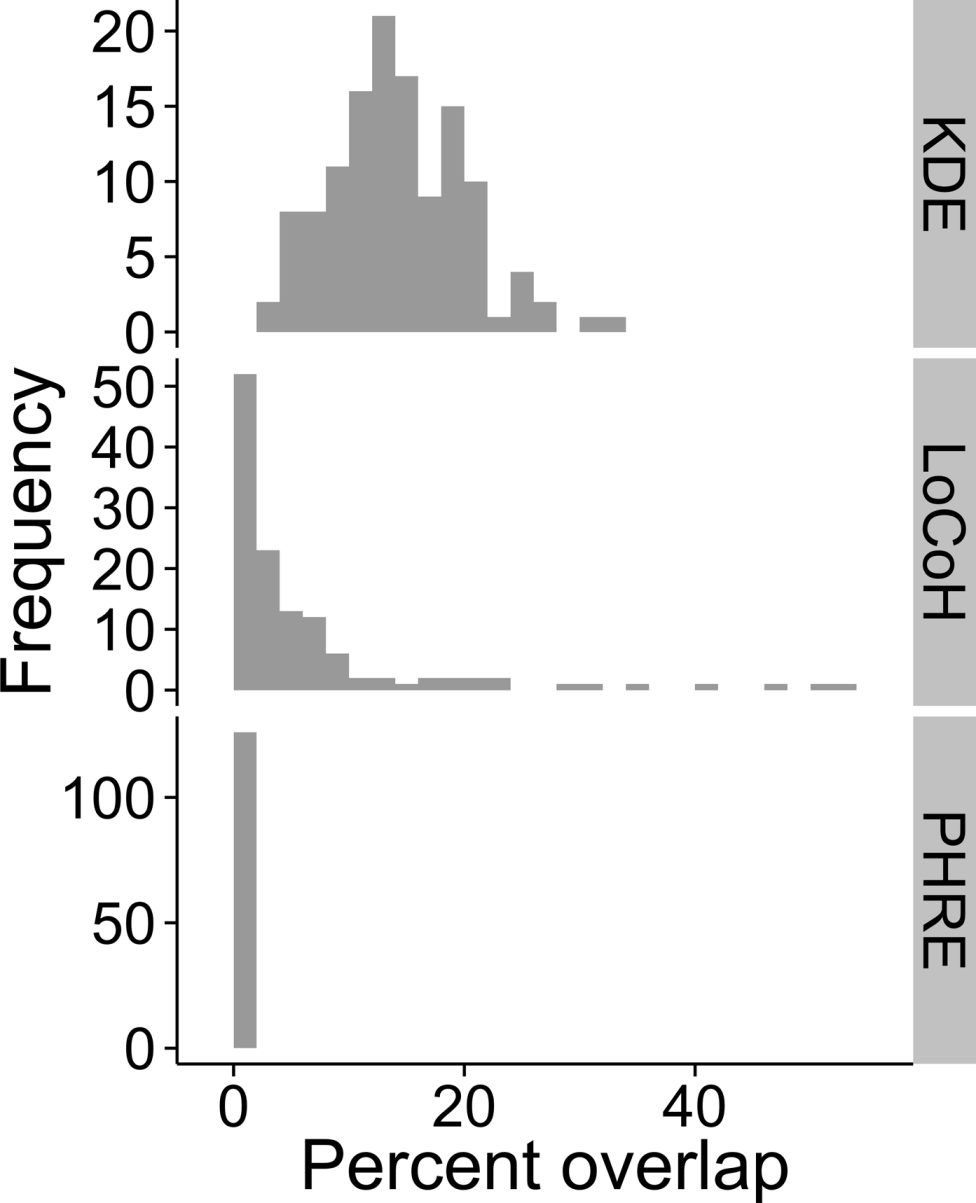
Frequency distribution of the percent of home range area that overlapped with land for each method. We calculated the percent of the 90% probability isopleth that overlapped with land (*N* = 126 sea otter home ranges for each method). Three methods are compared: KDE (top), LoCoH (center), and PHRE (bottom).

KDE resulted in home range estimates that overlapped the most with terrestrial habitat (13.59 [10.15, 18.10]% of home range area was on land; note that statistics are presented as median [quartile 1, quartile 3]), and LoCoH overlap was intermediate (2.67 [0.43, 7.30]%; Wilcoxon rank sum test of LoCoH versus KDE, *W* = 2321, *p* < 0.0001). At the extreme, the maximum overlap was higher for LoCoH (53.64%) than for KDE (32.22%), but more LoCoH home ranges (18 out of 126) completely excluded land than KDE home ranges (0 out of 126). PHRE home ranges completely avoided overlap with land, as the method defines the probability of unused areas as zero (Fig 2). Note that the precision of PHRE home range boundaries depends on the resolution of the grid across which the probability values are estimated, so minimal overlap (<1%) can result if the resolution of the estimation grid is lower than the resolution of the unusable habitat spatial data.

#### Sample size requirement

**Fig 3:**
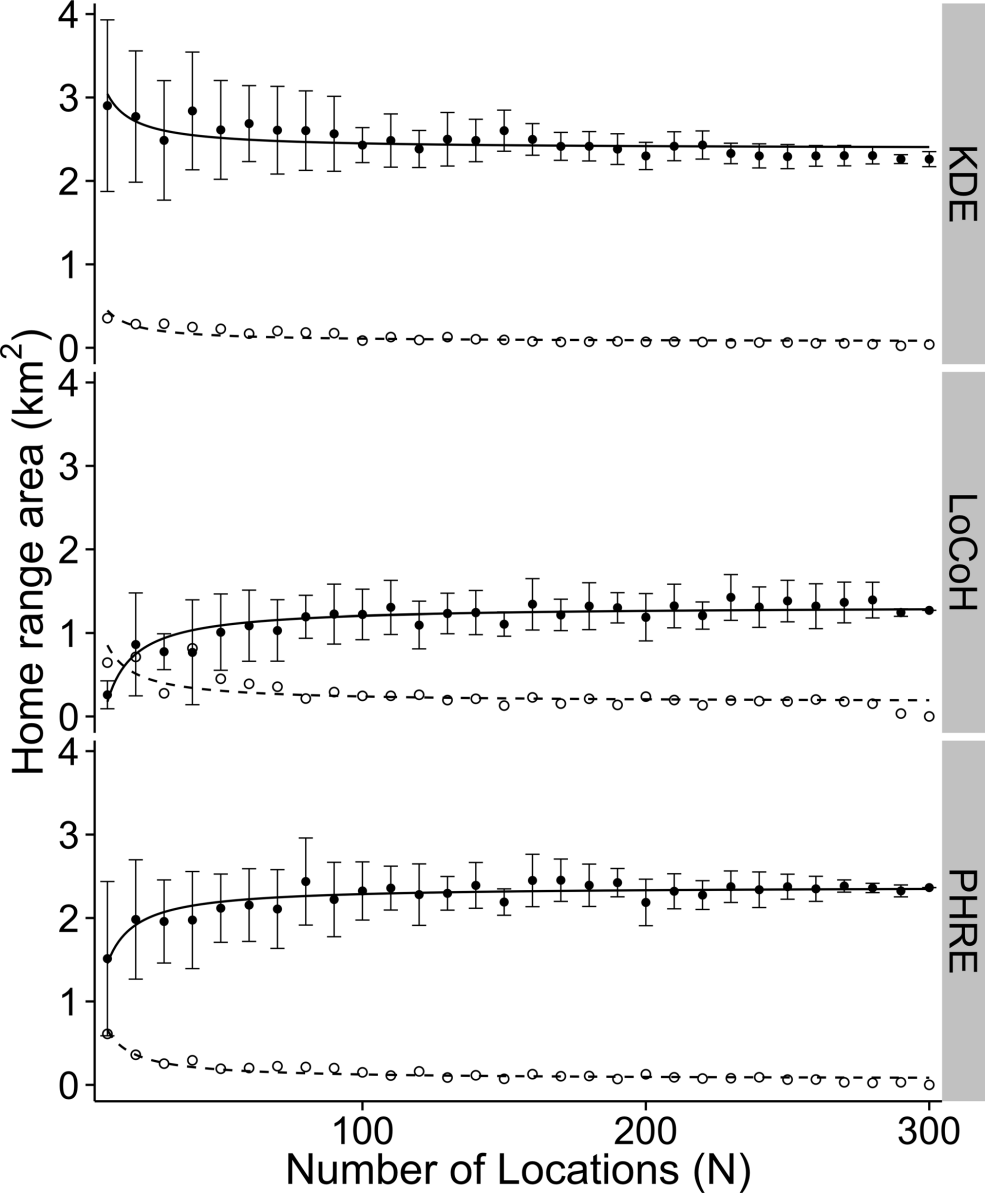
Average (± SD) home range area and the coefficient of variation across sample sizes of sighting locations. Home range estimates were iterated 10 times across each sample size for 26 different animals (data for only one animal shown, ID = N-1225-03-S). Closed circles show home range area and open circles show the coefficient of variation. The solid lines denote the asymptotic curves for the data and the dashed lines denote the asymptotic curves for the coefficients of variation. Three methods are compared: KDE (top), LoCoH (center), and PHRE (bottom).

By iterating home range estimates across sample sizes of sighting locations, we found that PHRE required the fewest sighting locations (*N =* 10 [10, 20]; chi-squared = 9.92, *df* = 2, *p* = 0.007; *W* = 417, *p* = 0.006). KDE required 50 (10, 80) sighting locations, while LoCoH polygons required 40 (10, 80) sighting locations. The requirements set by the coefficients of variation were statistically similar across methods (*df* = 2, *F* = 0.87, *p* = 0.42) and suggested using 210 (190, 230) sighting locations to minimize variation in estimated areas (Fig 3). Note that average area differed by method (log-transformed data were normally distributed, Kolmogorov-Smirnov test, *p* = 0.72; one-way ANOVA and Tukey HSD, *df* = 2, *F* = 27.53, *p* <0.0001), with PHRE tending to produce the largest home ranges (4.10 [2.29, 7.14] km^2^), KDE producing home ranges of intermediate area (3.23 [1.85, 5.04] km^2^), and LoCoH producing the smallest (1.81 [0.70, 3.41] km^2^). Area of the 90% polygon was sensitive to the smoothing parameter (which was not directly comparable across methods), so we withheld interpretation of polygon area and instead used a threshold independent analysis to address predictive accuracy of the probability estimates.

#### Predictive accuracy of probability estimates

**Fig 4:**
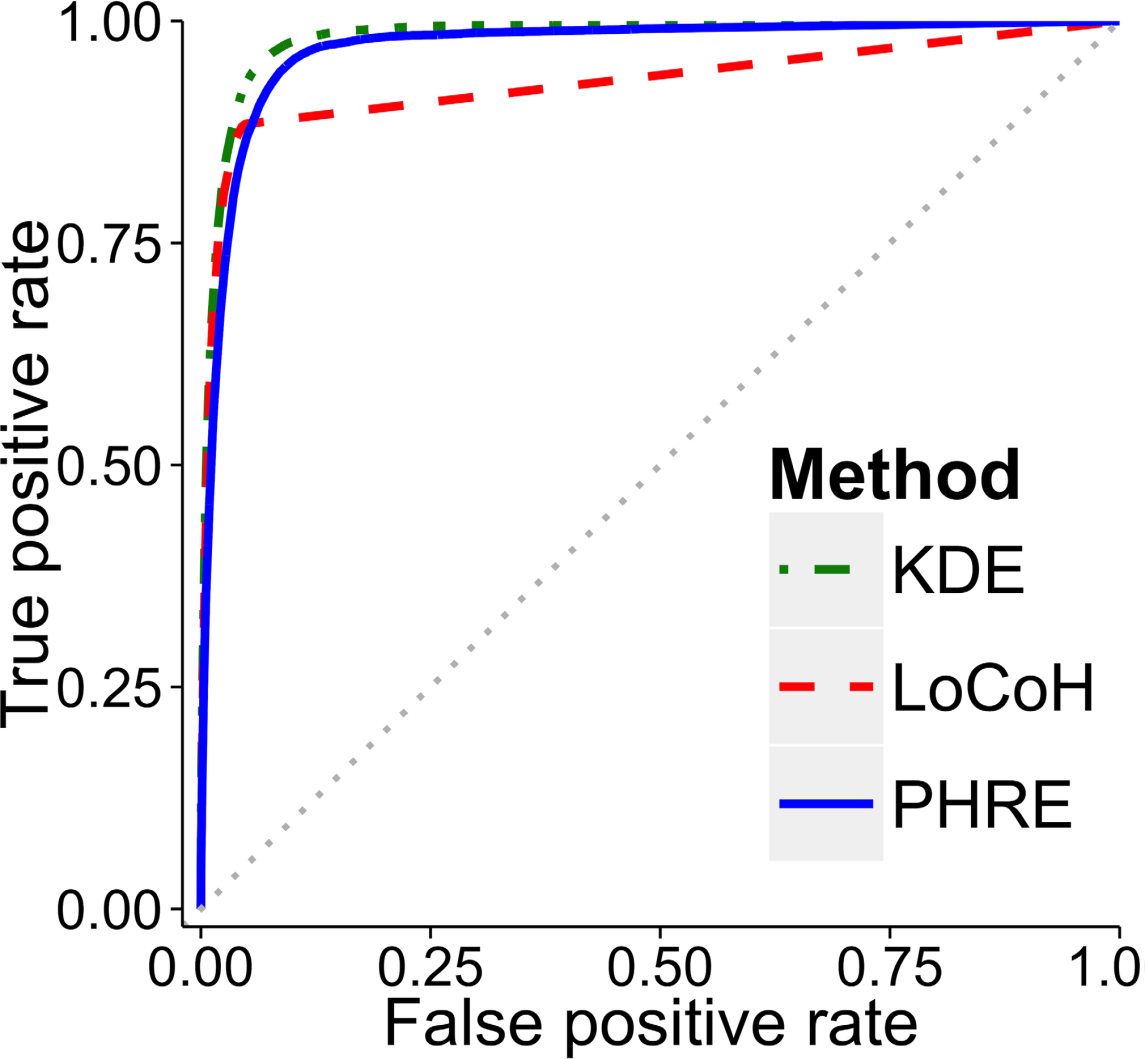
Receiver operating characteristic curves comparing predictive accuracy of KDE, LoCoH, and PHRE. Sighting data from 26 animals were used to generate home range estimates (200 random points were selected for ten iterations). For each iteration 100 presence and 1,000 pseudo-absence data were generated to calculate the area under the curve (AUC). Plotted curves show composite estimates for all iterations. Calculated curves can be compared to the grey dotted line, which denotes an AUC of 0.5 where presence and absence predictions are no better than chance.

All methods produced home range probability estimates that predicted locations of presence and pseudo-absence data better than chance (Fig 4). KDE and PHRE had high predictive accuracy (AUC = 0.98 ± 0.01 [mean ± standard deviation] and 0.97 ± 0.02 respectively), and LoCoH had lower predictive accuracy (AUC = 0.93 ± 0.02; *df* = 2, chi-squared = 454.26, *p* < 0.0001). Receiver operating characteristic curves showed that LoCoH displayed low true positive rates, indicating exclusion of used areas and negative bias (type I error). While KDE had the highest AUC values, the performances of KDE and PHRE were qualitatively similar, and both generally avoided negative (type I error) and positive bias (type II error).

### An Ecological Application

**Fig 5:**
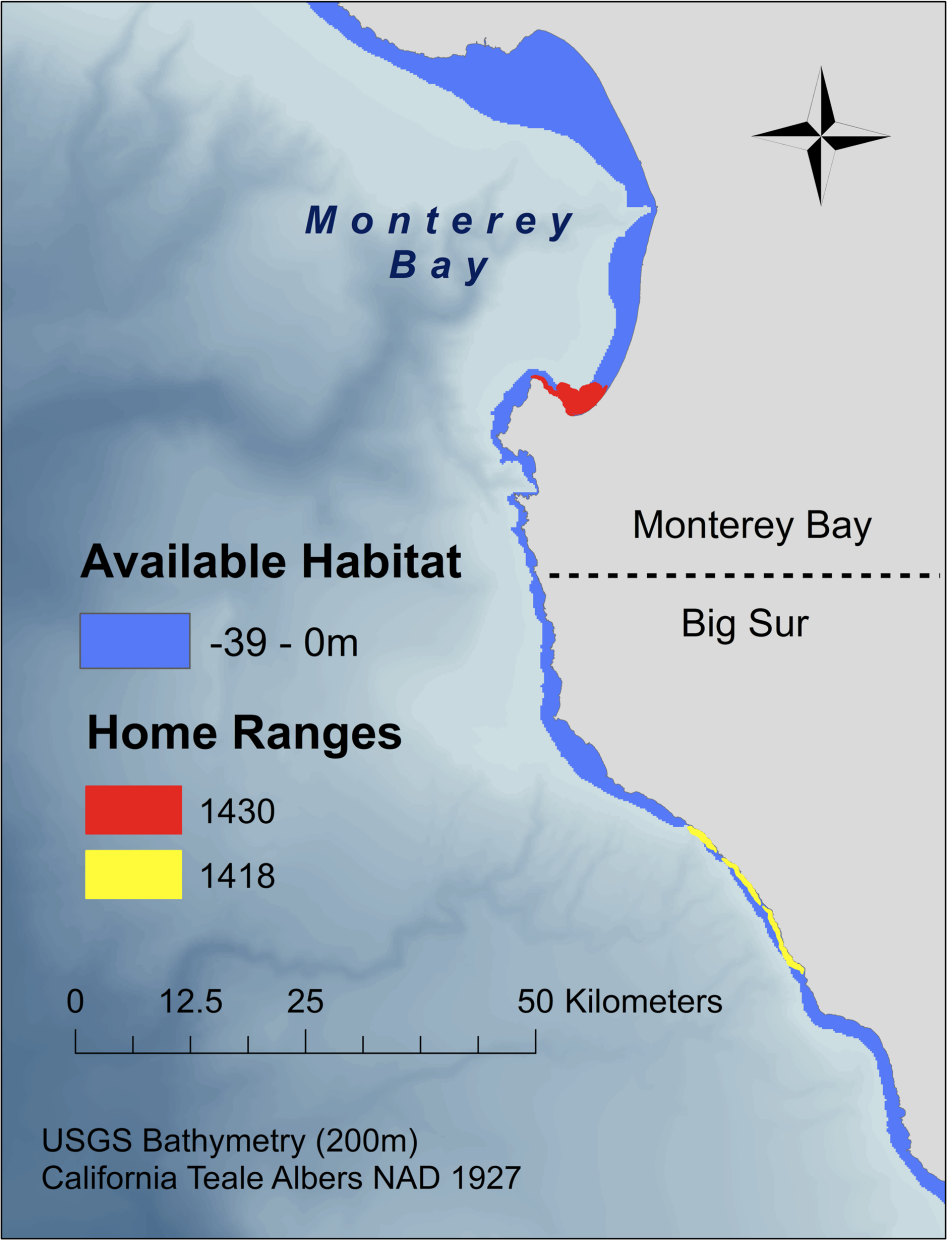
Map of available habitat at the Monterey Bay (north of Garrapata State Park) and Big Sur (south of Garrapata State Park) study sites. Because habitat available to foraging sea otters (between 0 and −39 meters depth) extends farther offshore in Monterey Bay, there is greater opportunity for sea otters to increase home range area and access to resources by extending home ranges offshore. In contrast, sea otters in Big Sur are forced to extend their home ranges along the coastline to access more resources. Characteristic home ranges for females at each study area are shown in red (Monterey Bay) and yellow (Big Sur).

**Fig 6:**
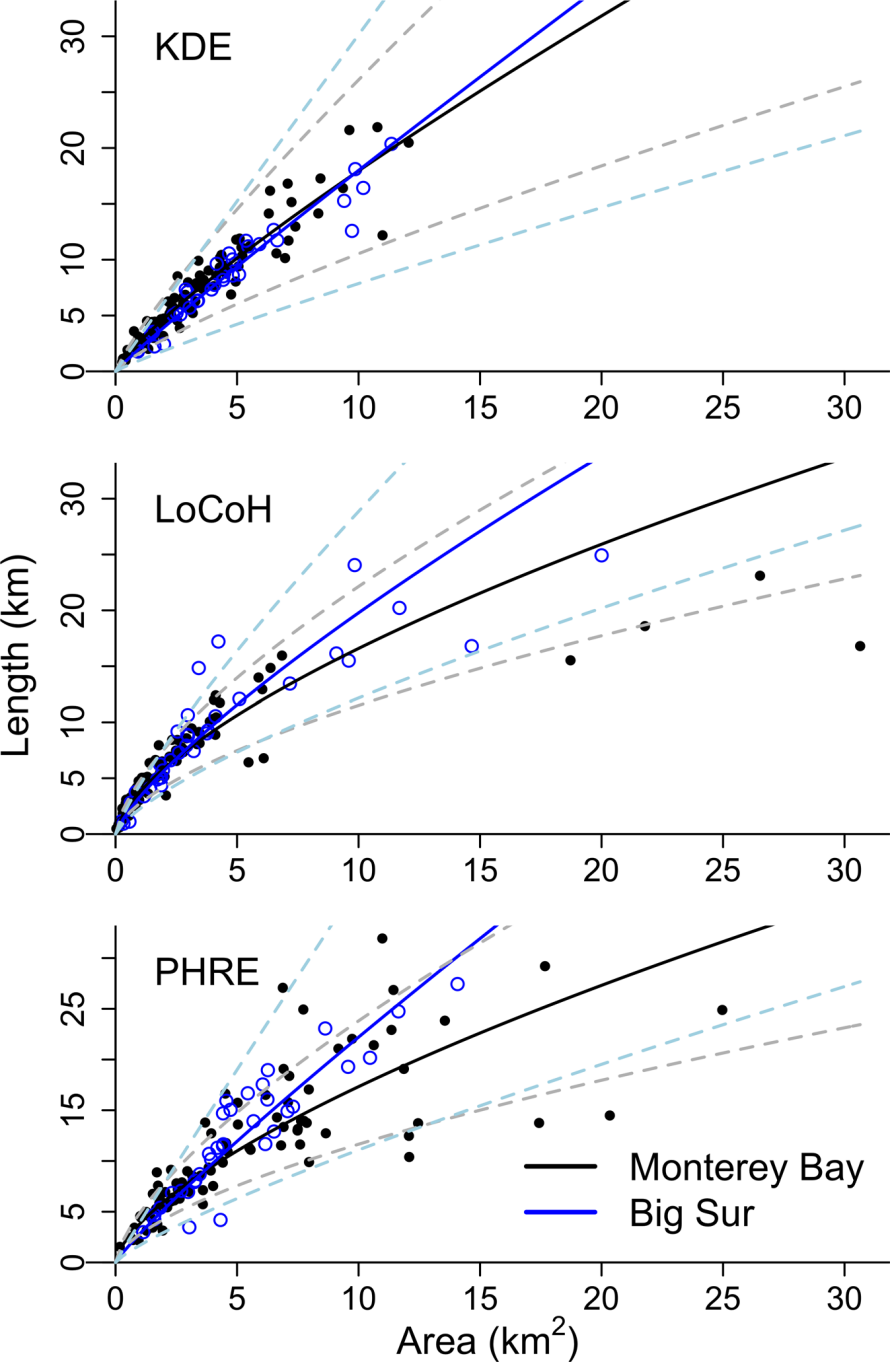
Home range length (km) plotted as a function of area (km^2^) for otters at two sites in central California. Data represent values obtained from home range estimates created using KDE (top), LoCoH (middle), and PHRE (bottom). Solid, black points represent Monterey Bay (*N* = 92) and open, blue points represent Big Sur (*N* = 34). Fitted power functions are shown by solid lines, with standard error shown by lighter dashed lines. Length increased more rapidly with home range area for sea otters at Big Sur compared to Monterey Bay (i.e. Big Sur home ranges were more elliptical).

Based on PHRE home range estimates, a typical 8.6-km stretch of coastline (the average home range length for otters in both habitats) contained 7.23 km^2^ of accessible area for benthic foraging in Monterey Bay and 5.10 km^2^ in Big Sur (Fig 5). To determine whether sea otter space use reflected these differences in available habitat, we compared home range shapes using the slopes of log-linearized functions representing the length-area relationship. The interaction term for the full linear model (Length ~ Area + Site + Area: Site) was statistically significant for KDE (*df* = 122, *t* = −0.12, *p* = 0.03), LoCoH (*df* = 122, *t* = −0.13, *p* = 0.01), and PHRE (*df* = 122, *t* = −0.24, *p* = 0.01), indicating that the assumption of slope homogeneity was not supported (Fig 6). Based on the magnitude of the difference between slopes of the linear models (and therefore the difference in home range shapes between sites), PHRE showed the largest effect size, where home range length was greater in Big Sur than in Monterey Bay (difference in slope coefficients = 0.24 ± 0.11 for PHRE, and 0.12 ± 0.06 and 0.13 ± 0.06 for KDE and LoCoH respectively). To interpret the biological significance of this difference, we calculated the expected difference in home range length in Big Sur versus Monterey Bay. Our analysis using PHRE indicates that a home range of average area (5.30 ± 4.24 km^2^) is 1.16 km longer in Big Sur compared to Monterey Bay. A home range of maximum area for Big Sur (14.07 km^2^) is 8.47 km longer than the equivalent home range in Monterey Bay.

## Discussion

Methods of describing animal space use have improved in their ability to incorporate complex environments. Non-parametric kernel estimation methods require fewer assumptions about patterns of animal space use than do parametric density functions or minimum convex polygons [29,52]. Local Convex Hull analysis [19] allows for the identification of moderately complex boundaries, and the lattice-based density estimator [53] allows for recognition of quite complex boundaries. However, while species distribution models use habitat variables to inform predicted distributions (see Guisan and Zimmermann [54] for a review), home range estimates rarely incorporate continuous habitat features a priori (but see Horne et al.’s [22] synoptic model of animal space use and Moorcroft et al.’s [24] Mechanistic Home-Range Analysis for a sophisticated application of this concept using movement models). Habitat selection analyses (such as maximum entropy [55] or general additive mixed models [56]) make use of such non-Cartesian dimensions, but they serve a fundamentally different purpose from home range analysis. PHRE is one of the first methods to directly incorporate continuous features of the environment in probability estimates of past space use by an individual. PHRE performed well in including used areas and excluding unused areas, allowing for meaningful statistical descriptions of home range use in complex, restricted habitats.

### Method Comparison

#### Exclusion of unused areas

PHRE proved to be more successful at excluding terrestrial areas than the other two home range methods. This is perhaps not surprising, given the methods of coordinate transformation (PHRE disallows any overlap with land, as unused areas receive a probability estimate of zero by definition). This particular feature of PHRE will be extremely useful for coastal-dwelling marine species such as sea otters, or other species where a complex “hard boundary” needs to be accommodated in home range methods. PHRE was more successful at this task than LoCoH, which also recognizes hard boundaries in animal space use [19]. However, PHRE requires that the feature(s) of unusable space be identified a priori, whereas LoCoH can highlight such boundaries with no a priori information. Thus if the goal is to estimate home ranges when there is suspicion of hard boundaries (but no means to pre-identify unusable space), then LoCoH would be a more suitable method, but if there are complex boundaries separating usable space from unusable space that is already identified (e.g. land vs. water, forest vs. non-forest) then PHRE may be the more effective method.

#### Sample size requirement

Depending on methods and species, home range estimates require sample sizes from 18 [57] to 1,000 [58]. For many species direct field observation is the only available approach for tracking space use, as satellite tags are only effective for wide ranging species (locations are of limited accuracy) and can be prohibitively expensive. As collecting sighting data using radio telemetry is labor intensive, requiring relatively few sighting locations is a desirable trait for a method that is applicable across systems. We found moderate differences in sample size requirements across methods. KDE and LoCoH required a median of 50 and 40 points respectively for home range area to approach an asymptote, whereas PHRE only required 10 points. Achieving stabilization of variance estimates required a larger sample size, approximately 210 sighting locations for all three methods. Note that when applying any of these methods to a new dataset, the exact sample size at which average home range area stabilizes depends on the distribution of sighting locations, so any estimate of sample size requirements will likely be system or species specific. In addition, our estimates of sample size requirements were made relative to a baseline of 300 sighting locations, which reflected sample sizes for animals that were well sampled over a two-year period in our study. Although this analysis indicates that the methods perform well at low sample sizes, we note that these sample size requirements only pertain to asymptotic estimates of area contained, and not to accuracy of home range shape and location. Thus, although lower sample sizes are required by PHRE to estimate the area enclosed within the home range boundary, this does not mean that home range shape and location are also stable at these lower sample sizes.

#### Predictive accuracy of probability estimates

An accurate method of home range estimation can identify and therefore predict both used and unused locations. Calculating the AUC for each method revealed that KDE predicted the location of presence and pseudo-absence data with the most accuracy (though only marginally better than PHRE). This result was somewhat surprising, as KDE performed the worst in the overlap test, so we expected that the method would suffer from low false positive rates (indicating inclusion of unused areas). We can reconcile this difference by reviewing how the “pseudo-absence data” were produced in the AUC analysis. Because pseudo-absence points were drawn randomly from any location within 1 km of the sighting locations, the absence data fell on both water and land. This fact leads to the somewhat paradoxical result that the KDE home ranges had the highest rate of exclusion of pseudo-absence points, because they were able to exclude both marine *and* terrestrial points. This highlights the fact that the AUC comparison should be qualified by the “exclusion of unusable space” comparison, as poor performance on the latter metric actually allowed for better performance on the former.

Of the three methods tested, PHRE was most successful at excluding unused areas, required the fewest sighting locations, and had high predictive accuracy. However, PHRE also comes with the challenge of obtaining both habitat data and determining species habitat requirements a priori. The preferred method for a given species will thus depend on habitat complexity at the scale of animal space use (to the degree that it influences the risk of type I and II error), availability of sighting data [10], availability of environmental data, and the degree to which researchers can identify habitat requirements a priori. For an animal in a relatively unrestricted, simple landscape, such as an African buffalo (*Syncerus caffer*) on a plain with uniform, high quality foraging opportunities [59], there is little risk of including unused areas (type I error), so KDE or LoCoH are preferred as they do not require habitat data. Although there is a risk of excluding used areas (type II error), LoCoH can be useful for describing home ranges in species that encounter moderately complex boundaries, such as white-faced capuchins (*Cebus capucinus*) in forested areas that avoid large clearings and grasslands [60], and have incomplete or coarse habitat data available. For species that inhabit a restricted and complex environment with a high risk of incorrectly including unused areas in home range boundaries, such as black bears (*Ursus americanus*) that avoid circuitous roads [61], northern pike (*Esox lucius*) in riverine habitats [21], and Arctic foxes in oil-developed areas with 50% of the land surface covered by water [62], PHRE provides a powerful new method for creating estimates that exclude these unused areas. PHRE should perform well in both aquatic and terrestrial environments and for other movement types (such as central-place foragers, where distance from the nest or den site could be included as an environmental feature), but this remains to be tested.

In addition to excluding unused areas and requiring few sighting locations, PHRE allows researchers to evaluate the fit of home ranges estimated using multiple habitat features. As PHRE can be performed in multi-dimensional space, alternative models incorporating different habitat variables can be tested against each other. For example, we found that sea otter home ranges predicted by coastal position and water depth were equally or more accurate than those predicted by coastal position and offshore distance (AUC using depth = 0.98 ± 0.01). In these endeavors, it is useful to have at least one feature that is grounded in geographic coordinate space with 1:1 mapping (e.g. distance along a boundary). Note that as more dimensions are added, alternative methods may be required to select the smoothing parameters [63]. When using more than two dimensions, we found that the reference smoothing parameter in the ks package provided a good visual fit to the data. By calculating the AUC, researchers can compare the accuracy of multiple models [64] that incorporate different habitat variables and choose those that are most biologically appropriate for their study species. While not a substitute for a habitat selection analysis, PHRE could serve as a superior null model in Horne et al.’s synoptic model [22], which would allow for subsequent interpretation of habitat selection.

### An Ecological Application

In applying PHRE to test the effect of habitat structure on home range shape, we expected that site bathymetry (a proxy for the distribution of accessible resources) would influence sea otter space-use. In support of our hypothesis, analyses showed that for a given home range area, length was greater in Big Sur than in Monterey Bay. Big Sur home ranges were therefore more elliptical overall, and this difference in home range length between sites increased as overall size increased. Mirroring their narrower continental shelf, Big Sur sea otters are only able to increase benthic foraging area by extending their home ranges farther along the coastline. In contrast, Monterey Bay sea otters are able to access shallow offshore resources, and thus can increase home range area by extending their home ranges farther offshore.

Differences in habitat and home range shape have implications for sea otter health, as home ranges of equivalent area are up to 8.47 km longer in Big Sur compared to Monterey Bay. Home range shape may influence risk of exposure to terrestrial pollutants, including zoonotic protozoan pathogens such as *Toxoplasma gondii*, which may be transported into marine ecosystems via sewage systems and freshwater runoff [65,66]. Encountering longer stretches of coastline in Big Sur may increase exposure to freshwater outputs from multiple watersheds and increase risk of encountering terrestrial pollutants for individual sea otters. In addition, otters with similar home range lengths are expected to realize up to a 37% loss of foraging habitat in Big Sur compared to Monterey Bay. While these restrictions could be offset by higher prey density in Big Sur, recent work on sea otter body condition and foraging success suggests that prey resources are equally or less abundant in Big Sur as compared to Monterey Bay [27].

The effect of habitat structure on home range shape has implications for costs and benefits of home range use across habitats. It is therefore important to note that the effect of site on home range shape was most detectable using PHRE. KDE, the method used in previous publications that define sea otter home ranges [67,68], showed a difference between sites of lower magnitude and less significance (Fig 6). Similarly, the effect size detected using LoCoH was half that of PHRE. Using PHRE to detect differences in home range shape will allow researchers to better evaluate space use trade-offs for species in complex habitats, such as sea otters. Accurate estimates of home range shape and location were previously unavailable for many species in restricted habitats; PHRE fills this niche, and has potential applications for research on exposure to anthropogenic disturbances, encounter rates with pathogens, and access to resources.

### Conclusion

PHRE performed well in the coastal environment by successfully excluding unused areas from home range polygons, displaying low sample size requirements, and creating probability estimates with high predictive accuracy and low bias (minimizing both type I and II errors). This method is applicable to ecological studies of species whose home ranges are restricted by complex boundaries or across environmental gradients. Limitations to this method include the need for environmental data and a priori knowledge of habitat features that influence animal space use. In systems for which these requirements are met, PHRE can provide more accurate home range estimates for species in restricted habitats than previous methods, leading to more realistic characterization of the physical and biotic environments with which an individual interacts. Increased accuracy in defining home ranges will allow researchers and resource managers to better understand habitat use requirements and ultimately improve conservation efforts for a variety of species.

## Supporting Information

S1 Fig Step 1 of Permissible Home Range Estimation: Sighting locations of an individual animal are collected over a set time period. Sighting locations of sea otter 1317, a female in Monterey Bay, CA, over a two-year period (2007–2009). Data were collected using VHF radio-telemetry. Projection: CA Teale Albers, NAD 1927.
(TIFF)

S2 Fig Step 2 of Permissible Home Range Estimation: Sighting locations are transformed from geographic coordinate space to landscape space. For sea otters, we assigned coastal position (ATOS) and distance from shore values to sighting locations. ATOS (As The Otter Swims) points are numbered sequentially and run along the 10-m isopleth at 500-m intervals (black points and numbers). Sighting locations (yellow points) are each assigned an ATOS value (yellow numbers) based on their proximity to ATOS points and a distance-from-shore value based on their distance to the closest point on land (vector along the red arrows).
(TIFF)

S3 Fig Step 2 continued: Sighting locations are transformed from geographic coordinate space (left) to landscape space (right). (TIFF)

S4 Fig Step 3 of Permissible Home Range Estimation: A kernel density function is generated in landscape space. Black points denote ATOS and *log*(distance) values of the sighting locations. Warmer colors indicate increasing density values.
(TIFF)

S5 Fig Step 4 of Permissible Home Range Estimation: Kernel density estimates are back-transformed to geographic coordinate space and converted to probability estimates. Using the kernel density function, density values are calculated for each point in a regularly spaced array along the central California coast. All kernel density values in the array are transformed to sum to one and reflect probability values. Projection: CA Teale Albers, NAD 1927.
(TIFF)

S6 Fig Step 5 of Permissible Home Range Estimation: Array points within the 90% probability kernel are selected and converted to a polygon, which defines the boundaries of the permissible home range. Grid points with probability values within the 90% probability kernel are selected and converted to a polygon to define a permissible home range. Projection: CA Teale Albers, NAD 1927.
(TIFF)

S1 File R code for generalized PHRE function. The function can be applied to any animal locations (dataframe) and habitat layers (rasters). Code was created in R version 3.0.2.
(R) Click here for additional data file.

S2 File Sample R code to apply the PHRE function to the sea otter dataset (see Data Accessibility Statement and S3 File). The user must open the R code file and specify the file paths to the data and the S3 File. Code was created in R version 3.0.2.
(R) Click here for additional data file.

S3 File Habitat rasters to use for S2 File. This.csv file contains an array of points (spaced 100 meters apart) along the California coast with ATOS values (ATOScal) and distance-from-shore values (Distance [m]). TealeX and TealeY coordinates are projected in California Teale-Albers NAD 1927. The depth (m) at each point is also provided. The S1 code converts this.csv file into raster layers to define the “landscape space” for Permissible Home Range Estimation in sea otters.
(CSV)
