# Supplementary figures and images for: Permissible Home Range Estimation (PHRE) in Restricted Habitats: A New Algorithm and an Evaluation for Sea Otters
Source: PLoS One. 2016 Mar 22;11(3):e0150547. doi: 10.1371/journal.pone.0150547 (PMC4803229; doi:10.1371/journal.pone.0150547)

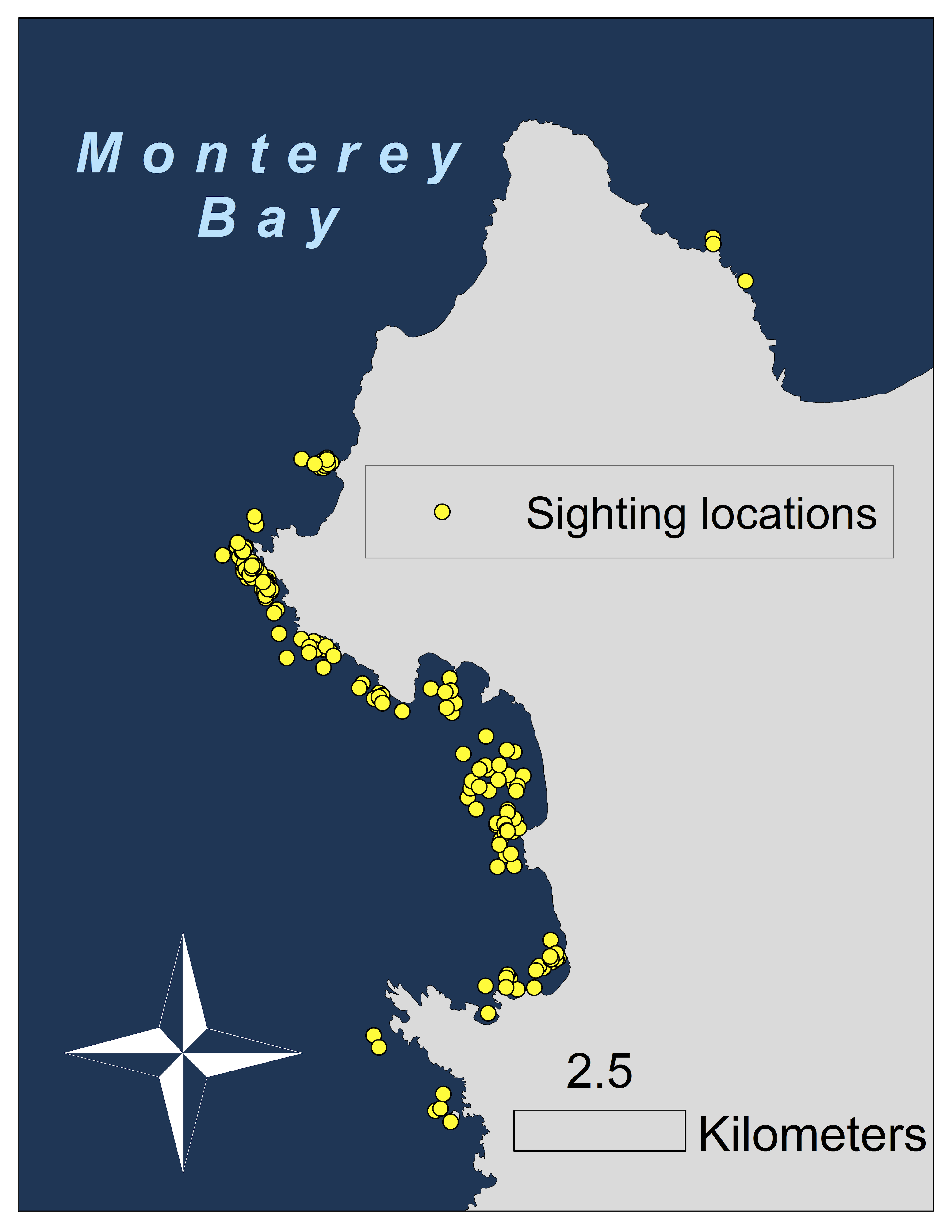

Supplement: S1 Fig — Sighting locations of sea otter 1317, a female in Monterey Bay, CA, over a two-year period (2007–2009). Data were collected using VHF radio-telemetry. Projection: CA Teale Albers, NAD 1927. (TIFF) [file pone.0150547.s001.tiff]

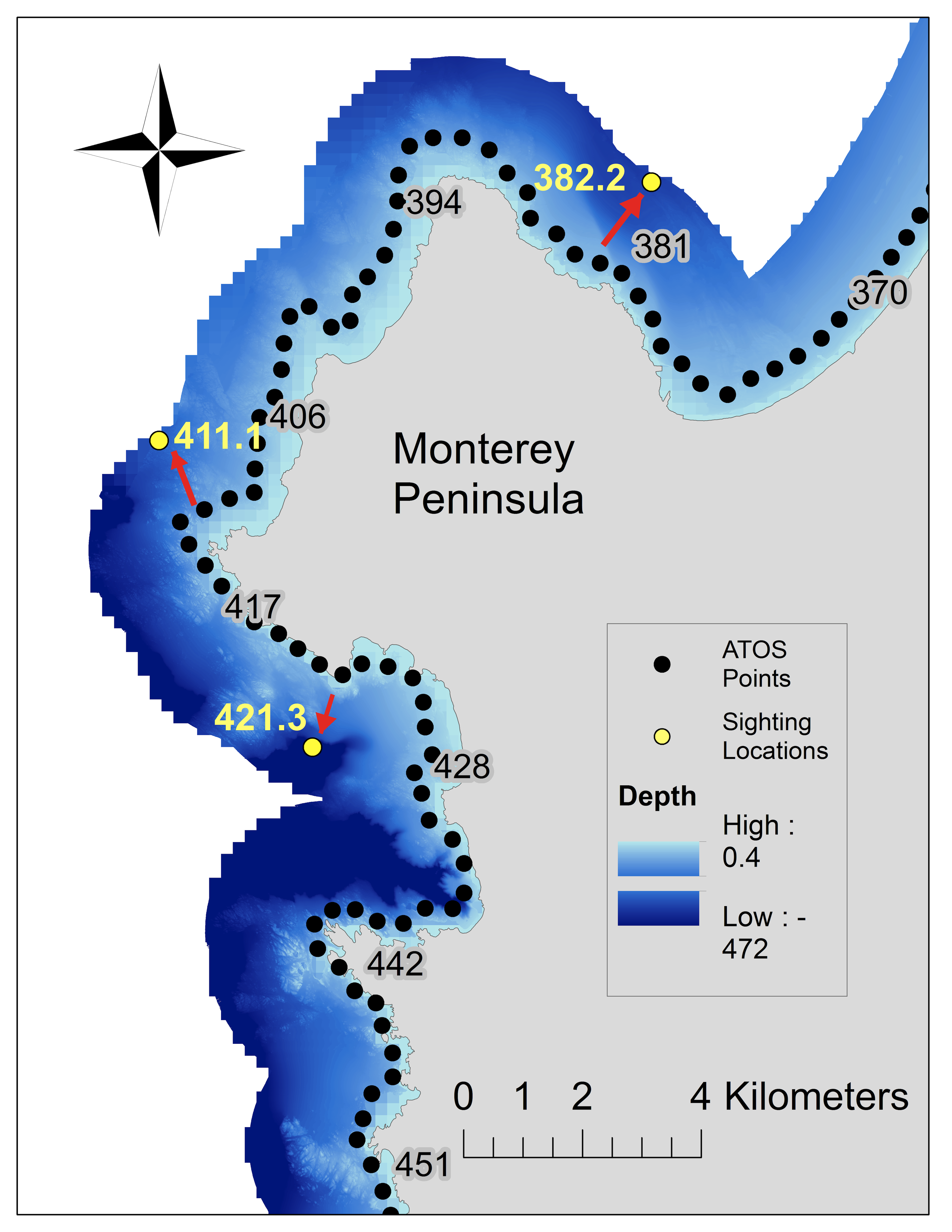

Supplement: S2 Fig — For sea otters, we assigned coastal position (ATOS) and distance from shore values to sighting locations. ATOS (As The Otter Swims) points are numbered sequentially and run along the 10-m isopleth at 500-m intervals (black points and numbers). Sighting locations (yellow points) are each assigned an ATOS value (yellow numbers) based on their proximity to ATOS points and a distance-from-shore value based on their distance to the closest point on land (vector along the red arrows). (TIFF) [file pone.0150547.s002.tiff]

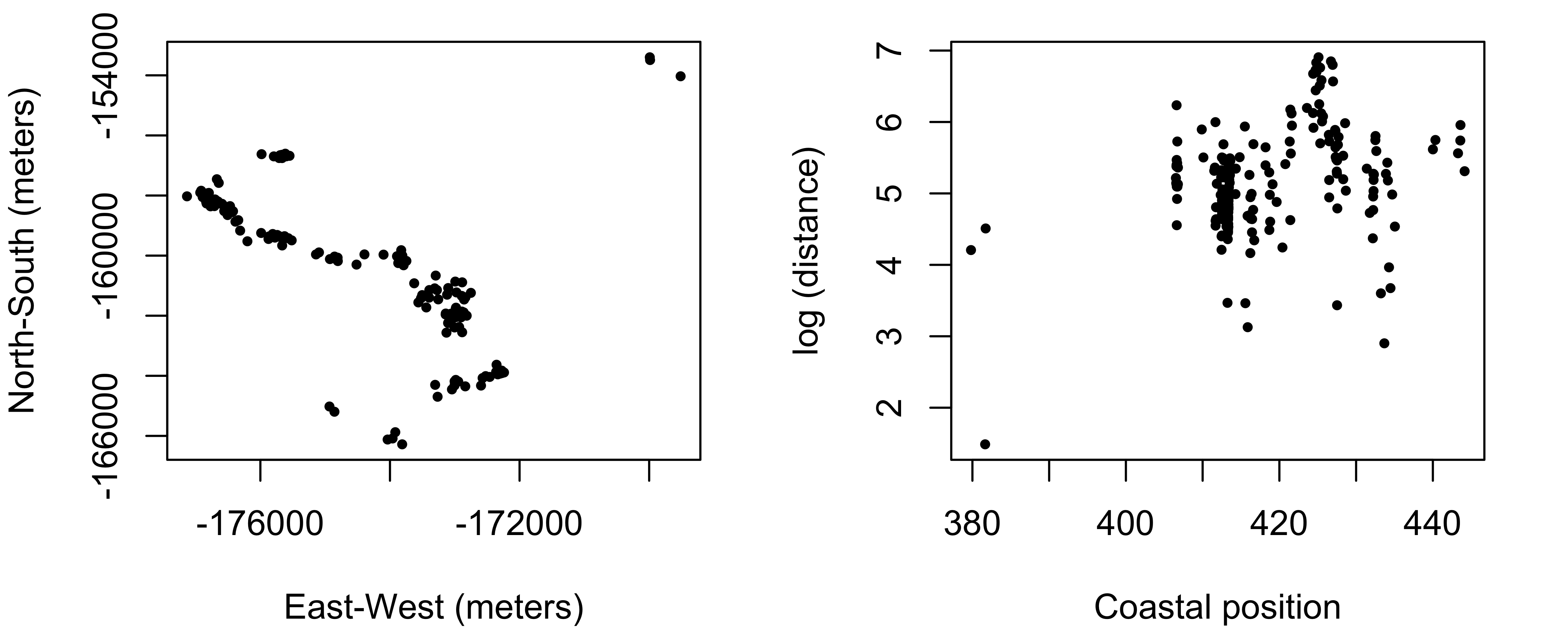

Supplement: S3 Fig — (TIFF) [file pone.0150547.s003.tiff]

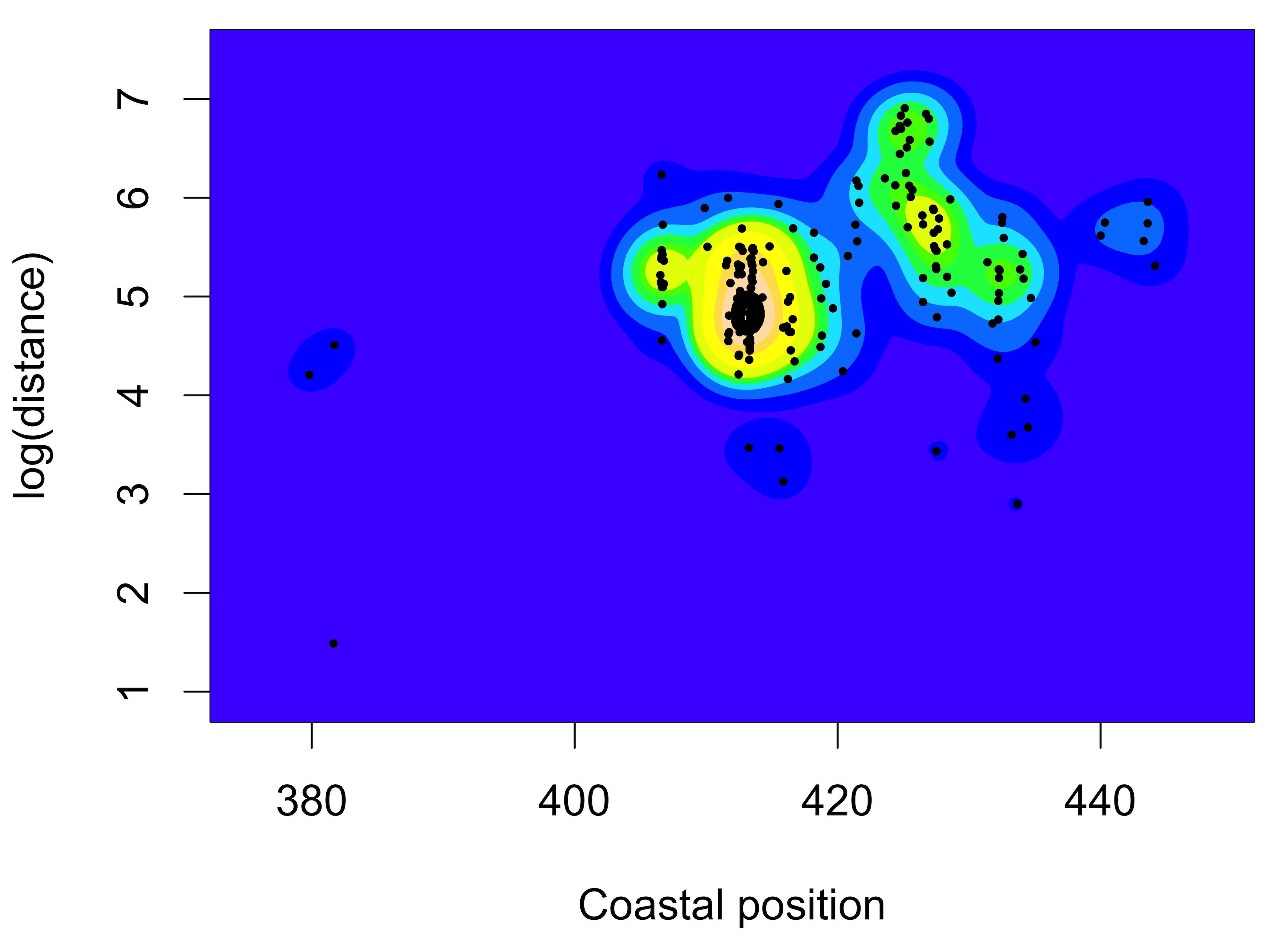

Supplement: S4 Fig — Black points denote ATOS and log(distance) values of the sighting locations. Warmer colors indicate increasing density values. (TIFF) [file pone.0150547.s004.tiff]

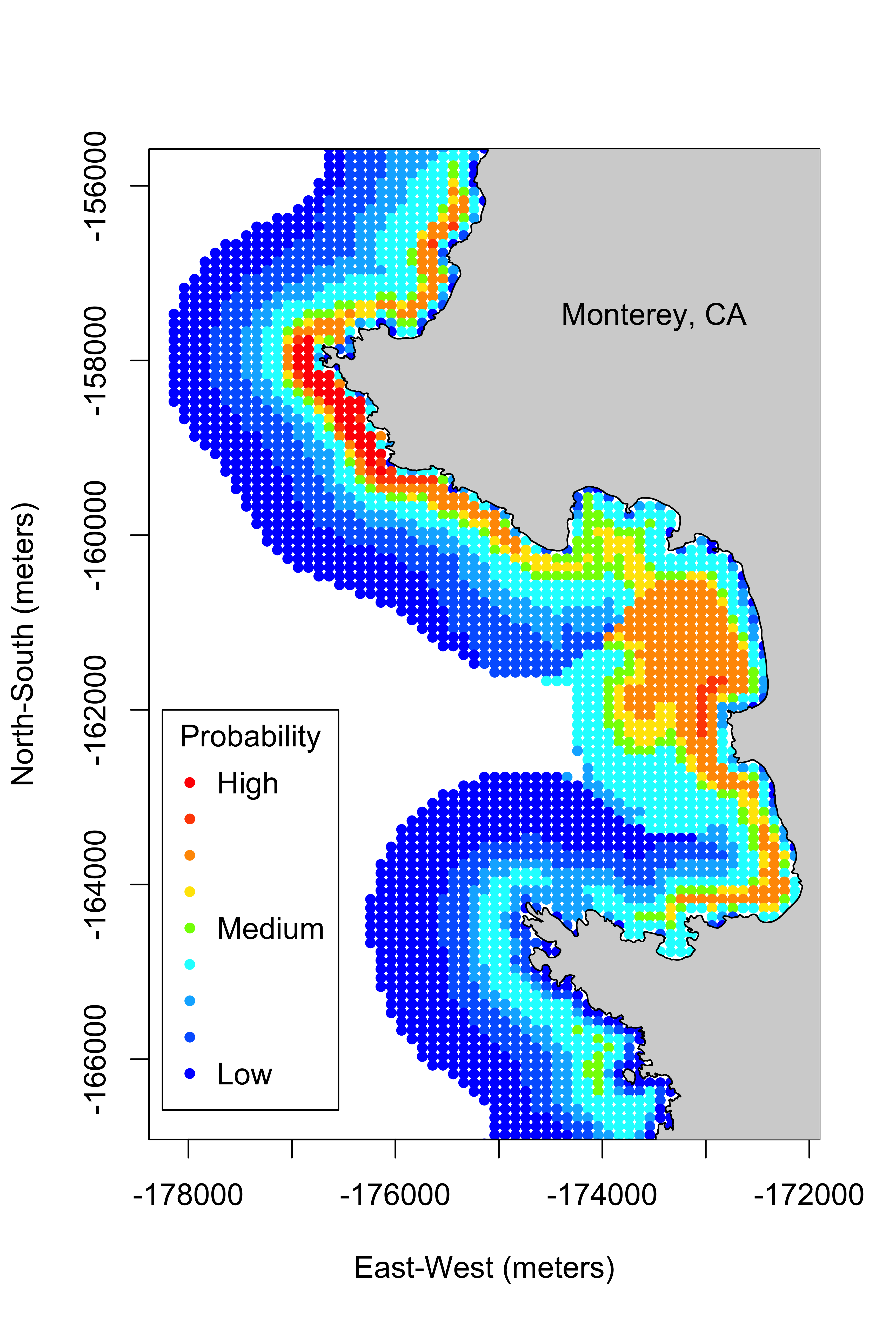

Supplement: S5 Fig — Using the kernel density function, density values are calculated for each point in a regularly spaced array along the central California coast. All kernel density values in the array are transformed to sum to one and reflect probability values. Projection: CA Teale Albers, NAD 1927. (TIFF) [file pone.0150547.s005.tiff]

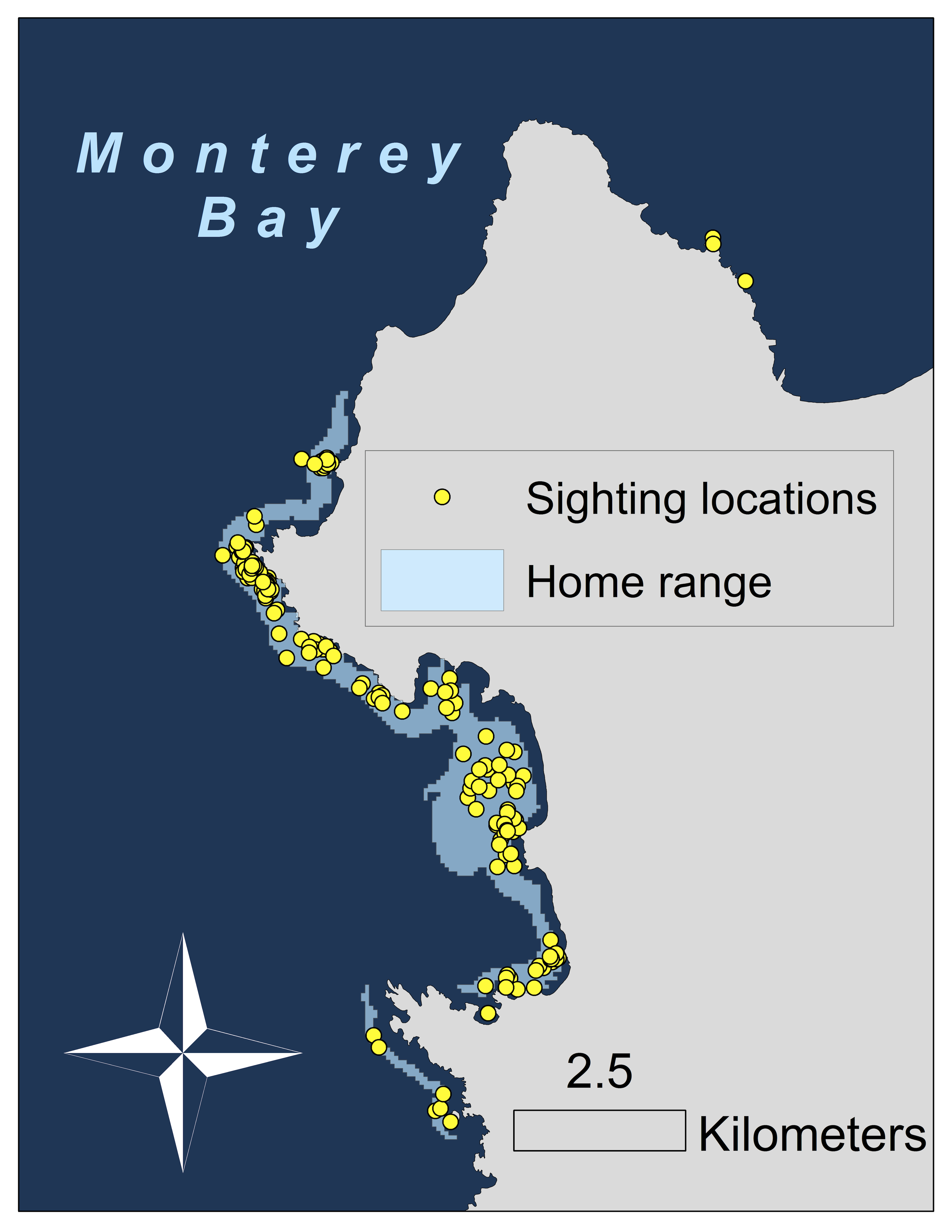

Supplement: S6 Fig — Grid points with probability values within the 90% probability kernel are selected and converted to a polygon to define a permissible home range. Projection: CA Teale Albers, NAD 1927. (TIFF) [file pone.0150547.s006.tiff]
